# Editorial: Genetic and molecular determinants in bone health and diseases - volume II

**DOI:** 10.3389/fendo.2026.1789444

**Published:** 2026-01-27

**Authors:** Michela Rossi, Andrea Del Fattore

**Affiliations:** Bone Physiopathology Research Unit, Translational Pediatrics and Clinical Genetics Research Division, Bambino Gesù Children’s Hospital, IRCCS, Rome,, Italy

**Keywords:** bone diseases, bone health, epigenetic, genetic bone diseases, molecular pathways

In recent years, advances in genetics, multi-omics technologies, and molecular biology have profoundly transformed our understanding of bone biology. The skeleton is now recognized as a highly dynamic organ whose development, remodeling, and pathological alterations are governed by complex and finely regulated genetic programs and molecular networks (1–3). Large-scale genomic studies have identified hundreds of loci associated with bone mineral density and fracture risk, revealing the polygenic architecture of common skeletal traits and diseases (4, 5). New techniques, such as transcriptomic, proteomic, and metabolomic have enabled the systematic characterization of cellular pathways that regulate osteoblast and osteoclast differentiation, activity, and survival (6, 7). Beyond genetic variation, epigenetic mechanisms and microRNA have emerged as key regulators of skeletal cell fate and function, modulating gene expression in response to development, mechanical loading, aging, and disease-associated factors (8, 9). Importantly, the integration of multi-omics datasets with clinical phenotypes is accelerating the identification of biomarker and the development of targeted and personalized new therapeutic strategies (10, 11).

The Research Topic *Genetic and Molecular Determinants in Bone Health and Diseases – Volume II* brings together ten contributions that explore bone health and disease through the lenses of genomics, transcriptomics, proteomics, epigenetics, and translational medicine.

## Genetic architecture of skeletal disorders

The genetic involvement of rare skeletal disorders is one of the major focuses of this Research Topic.

Najjar et al. addressed the complexity of skeletal dysplasias within a national health system framework, highlighting how genetic heterogeneity underlies the wide clinical spectrum of these rare conditions. Their work describes the importance of integrating molecular diagnostics into routine clinical practice to improve disease classification, prognosis, and patient management.

Similarly, Portales-Castillo et al. reviewed disorders caused by homozygous mutations in *PTH1R*, a key gene in bone and mineral metabolism. By delineating genotype-phenotype correlations, they provide insights into the molecular mechanisms by which alterations in PTH signaling disrupt skeletal development and homeostasis, paving the way for more targeted therapeutic interventions.

Pagliarosi et al. explored the emerging genetic alterations associated with Gorham-Stout disease, a rare osteolytic disorder with unknown etiology. By integrating available genetic and molecular data, the authors discussed the potential involvement of angiogenic, lymphangiogenic, and osteoclastogenic pathways in progressive bone loss. Their review provides a mechanistic framework for future functional studies and highlights the relevance of molecular genetics in deciphering ultra-rare bone diseases.

In the work of Schembri and Formosa, a focus was performed on the identification of osteoporosis genes using family-based studies. They reviewed the evidence showing how rare, high-impact variants segregating within families can uncover novel genetic determinants that may be missed by population-based genome-wide association studies. Their work reinforces the translational potential of genetic profiling for risk stratification and personalized approaches to osteoporosis prevention and management.

## Multi-omics approaches and systems biology

In the work of Wang et al. diabetic sarcopenia was investigated using a proteomic strategy, revealing that dysregulation of autophagy- and apoptosis-related pathways contributes to muscle deterioration in diabetes. Although focused on muscle, their findings are highly relevant to skeletal biology, as these same molecular programs influence osteoblast and osteoclast survival and activity. This study highlights how omics-based profiling can identify shared molecular determinants of musculoskeletal decline and opens new perspectives for integrated therapeutic strategies targeting both muscle and bone in metabolic disease.

Li et al. applied a multi-omics strategy to identify Meteorin-like (Metrnl/IL-41) as a novel regulator of bone metabolism and disease activity in ankylosing spondylitis. By integrating omics approaches the authors demonstrated how inflammatory signaling interfaces with molecular pathways regulating bone remodeling.

The intersection between bone and muscle biology, a growing area of translational relevance, is addressed by Yi et al. in their comprehensive review of osteosarcopenia. By integrating epidemiological data with molecular mechanisms, the authors depict osteosarcopenia as a systemic disorder driven by shared genetic and molecular pathways affecting both tissues. This integrative view exemplifies how molecular insights can redefine clinical syndromes and guide the development of combined therapeutic strategies targeting the bone–muscle unit.

## Epigenetics and non-coding RNA regulation

Epigenetic regulation and non-coding RNAs emerge as key determinants of bone health in several contributions.

Lin et al. provided a systematic review of the role of circular RNA and long non-coding RNA (ncRNA) in osteonecrosis of the femoral head. The authors highlighted how these epigenetic regulators modulate key biological processes, including apoptosis, angiogenesis, oxidative stress, and extracellular matrix remodeling. Their work emphasizes the importance of post-transcriptional regulation in skeletal pathology and suggests that ncRNA may represent both diagnostic biomarkers and therapeutic targets.

Moreover, Thakore and Delany examined microRNA-based regulation in growth plate cartilage, focusing on how specific miRNA control chondrocyte proliferation, differentiation, and hypertrophy. By dissecting the molecular mechanisms and downstream targets involved in longitudinal bone growth, the authors illustrated the central role of epigenetic regulation in skeletal development. This review also highlights the therapeutic potential of miRNA-based strategies for growth disorders and regenerative medicine.

## Genetics and translational implications for clinical practice

Zhou et al. reviewed the association between single nucleotide polymorphisms and susceptibility to osteomyelitis and prosthetic joint infection. Their analysis showed how host genetic variation influences immune responses, inflammatory pathways, and infection risk in skeletal tissues. These findings underscore the relevance of molecular genetics for patient stratification and for developing personalized strategies in orthopedic surgery and infection prevention.

## Conclusion

The studies included in this Research Topic demonstrated how genetic and molecular discoveries are increasingly translated into clinical contexts. From rare skeletal disorders to complex conditions such as osteoporosis, the integration of omics technologies, and epigenetic research is reshaping diagnostic and therapeutic paradigms. These contributions highlight the potential of molecular profiling to support precision medicine, enabling earlier diagnosis, refined risk assessment, and the identification of novel therapeutic targets.
